# Understanding of Constipation Symptoms and the Diagnosis and Management of Constipation in Chinese Physicians

**DOI:** 10.1371/journal.pone.0152801

**Published:** 2016-03-31

**Authors:** Huikuan Chu, Xiaohua Hou

**Affiliations:** Division of Gastroenterology, Union Hospital, Tongji Medical College, Huazhong University of Science and Technology, 1277 Jiefang Road, Wuhan, China; University Hospital Llandough, UNITED KINGDOM

## Abstract

**Background and Aims:**

Although a range of guidelines for the diagnosis and treatment of chronic constipation has been carried out, there was very little information about the understanding on constipation. The aim of the present study was to estimate the understanding of constipation symptoms and the diagnosis and management of constipation by clinical physicians in China.

**Methods:**

Participants were physicians and researchers in the field of gastroenterology in China who were scheduled to attend the National Conference on gastrointestinal motility(Constipation). Based on the recommendation of the Rome Foundation Board, the self-reported questionnaire was constructed.

**Findings:**

Although most of the opinions on symptoms of constipation were consistent, there were still some differences. Opinions on the Bristol stool form during constipation were discordant, 34% of the doctors thought that it was type 1 and type 2, while 46%of the doctors suggested that type 3 should also be considered constipation. There was no significant difference between them(P = 0.05); We investigated the interpretation on the duration of defecation prolonged, 27% of the doctors suggested it should be longer than 10 minutes, 22% of the doctors suggested it should be longer than 20 minutes, and other 22% of the doctors suggested it should be time of defecation became longer compared to previously bowel habits, there was no significant difference among them(P = 0.38).Only 36% of the doctors thought that psychotherapy was most important in the treatment of severe constipation, while 37% of the doctors thought that medication treatment was most important in the treatment of severe constipation, there was no significant difference between them(P = 0.895).

**Conclusion:**

We were able to obtain valuable information about current views on symptoms of constipation and the diagnosis and treatment of constipation among Chinese doctors. Although most of the opinions were consistent there were still some differences. This study indicated that in practice in China there was a need for further study on the role of constipation symptoms and there may also be a need for better establishment of consensus guidelines for constipation.

## Introduction

The prevalence of constipation ranged from 2% to 34%[1–5]. This variability may be attributed to the dietary habits[6, 7], socioeconomic status[8], regional differences[1,2], race[1,2], society[9], culture[9], et al. Besides, the different understanding on definition of constipation may be one of the important reasons. Although there are established diagnostic criteria such as Rome III[10–12], with the evolvement of definition on constipation, different researchers had different understanding on constipation, which led to discordant results.

First, prevalence rates of constipation were different with different definitions[5, 13–15]. Second, presence of constipation symptoms was different in different studies[5,16–18]. Furthermore, it was also reported that presence of symptoms was different with different definitions [13]. Third, although most of the physicians thought that presence of symptoms were different with different subtypes[10, 19], Koch et al.[20] reported that symptoms were not useful for differentiation among the pathophysiological subtypes of constipation. Fourth, treatment effects were different with different understanding on constipation. Conventional wisdom suggested that biofeedback treatment could improve electromyography values or symptoms[21, 22]. Recently, it was reported that biofeedback could improve both quality of life (QOL) and symptoms[23]. In a word, prevalence rates, categorizations and treatment effects of constipation were different with the discordant understanding of constipation.

Although a range of guidelines for the diagnosis and treatment of chronic constipation has been carried out[24–30], the definite understanding on constipation was still discordant and there was very little information about the understanding of constipation symptoms and the diagnosis and management of constipation in Chinese physicians. The aim of the present study was to estimate the understanding of constipation symptoms and the diagnosis and management of constipation by clinical physicians in China.

## Methods

Participants were physicians and researchers in the field of gastroenterology in China who were scheduled to attend the National Conference on gastrointestinal motility(constipation). This study was performed in March 2012.This experiment was approved by Huazhong University of Science and Technology Clinical Trial Ethics Committee (Wuhan, China).Methods used in this study were carried out in accordance with the approved guidelines and written informed consent was obtained from each participant.

Based on the recommendation of the Rome Foundation Board, the self-reported questionnaire was constructed. The questionnaire began with several general characters of physicians (e.g. type of hospital, position, working experience). The followed were items associated with symptoms, diagnosis and treatment of constipation. All of the items were single choice questions.

The following questions were included in the symptoms section: (1) Which was the most common symptom of constipation? (2) Which was the Bristol stool form during constipation? (3) How much force should be increased while straining? (4) How often was the frequency of defecation for infrequency of defecation? (5) How long should be spent when duration of defecation prolonged? (6) Complete spontaneous bowel movements (CSBM) were defined as: (7) Which was the most common symptom of slow transit constipation (STC)? (8) Which was the most common symptom of defecation disorders? (9) What was the difference between STC and defecation disorders? (10) What was the difference between irritable bowel syndrome with constipation (IBS-C) and functional constipation (FC)? From these answers, we could know about the understanding of constipation symptoms in Chinese doctors.

In order to get the information about diagnosis of constipation in China, the following questions were included: (1) Which was the most important factor for the diagnosis of STC? (2) Which was the most important factor for the diagnosis of defecation disorders?

At last, we wanted to know the management methods of constipation and designed the following questions: (1) Which was the most common risk factor of constipation? (2) Which was the major method to solve the problem of constipation? (3) Which was the most common risk factor of severe constipation(severe is defined as persistent symptoms with great suffering and affecting the quality of life, requiring continual administration of drugs[31])? (4) Which was the most important in the treatment of severe constipation? (5) Which categories of drugs was the most common used during the process of treating constipation? (6) Which was the most important in the treatment of STC? (7) Which was the most important in the treatment of defecation disorders?

Statistical data were analyzed using the SPSS package version 17.0 (SPSS, Chicago, IL, USA). The data were evaluated by Mantel-Haenszel chi-square test. A value of P < 0.05 was regarded as a significant difference for comparisons between groups.

## Results

A total of 148 attendees were investigated. Data were analyzed from 130 attendees excluding those who failed to respond. 61% of the physicians worked in reference medical centers, 38% of the physicians worked in primary care centers and 1% of the physicians worked in gastrointestinal specialized hospital.47% of the attendees were chief physicians, 25% were associate chief physicians, 14% were attending physicians, 4% were resident physicians and 10% were interns. Meanwhile, we investigated the working experience of the attendees, 75% were longer than 10 years, 15% were less than 5 years and 10% were 5–10 years.

### Understanding on symptoms of constipation

It was consistent in opinions on which was the most common symptom of constipation, and 61% of the attendees suggested it should be straining (Table 1),which was significantly higher than other questions (P<0.01). However, opinions on the Bristol stool form during constipation were discordant. 34% of the doctors thought that it was type 1 and type 2, while 46%of the doctors suggested that type 3 should also be considered constipation. There was no significant difference between them (P = 0.05).

**Table 1:** Understanding on symptoms of constipation.

which is the most common symptom of constipation	
straining	61%
lumpy or hard stools	15%
infrequency of defecation	16%
sensation of incomplete evacuation	4%
sensation of anorectal obstruction/blockage	1%
Time spend at defecation prolonged	3%
which was the Bristol stool form when constipation	
type 1 and type 2	34%
anyone among type1 to type3	46%
anyone among type1 to type4	10%
anyone among type1 to type5	4%
anyone among type1 to type6	2%
anyone among type1 to type7	4%
How much force should be increased when straining?	
<25%	2%
25%	15%
26%-50%	41%
51–75%	19%
>75%	4%
unclear	19%
How often was the frequency of defecation when infrequency of defecation?	
times of defecation decreased compared with previously	16%
less than 5 times per week	2%
less than 3 times per week	67%
less than 2 times per week	15%
unclear	0.00%
How long should be spent when duration of defecation prolonged?	
>5min	10%
>10min	27%
>20min	22%
>30min	17%
time of defecation became longer compared to previously bowel habits.	22%
unclear	2%
CSBM was defined as	
BM in which no laxative, enema, or suppository was used in the preceding 24 h, without a feeling of complete bowel emptying	94%
BM in which no laxative, enema, or suppository was used in the preceding 24 h, which was associated with a feeling of complete bowel emptying	3%
BM in which no laxative, enema, or suppository was used in the preceding 24 h	3%
unclear	0%
What was the difference between STC and defecation disorders?	
infrequency of defecation	6%
amount of stool decreased	3%
lack of defecation sensation	57%
difficult defecation	24%
unclear	10%
What was the difference between IBS-C and FC?	
Abdominal discomfort or pain with constipation, pain or discomfort symptoms disappeared after defecation	22%
Abdominal discomfort or pain with constipation, pain or discomfort symptoms relieved after defecation	12%
Abdominal discomfort or pain with constipation, pain or discomfort symptoms relieved or disappeared after defecation	59%
Abdominal discomfort or pain with constipation, pain or discomfort symptoms remained after defecation	3%
unclear	4%

41%of the doctors thought that straining as force should be increased 26%-50% (Table 1),which was significantly higher than other questions (P<0.01);67% of the doctors defined the infrequency of defecation as less than 3 bowel movements per week (Table 1), which was significantly higher than other questions (P = 0.011). We investigated the interpretation on the duration of defecation prolonged, 27% of the doctors suggested it should be longer than 10 minutes, 22% of the doctors suggested it should be longer than 20 minutes, and other 22% of the doctors suggested it should be time of defecation became longer compared to previously bowel habits, there was no significant difference among them (P = 0.38).

We also investigated the understanding about the definition of CSBM (Table 1) and 93% of the doctors thought that the CSBM was defined as a bowel movements (BM) in which no laxative, enema, or suppository was used in the preceding 24 h, which was associated with a feeling of complete bowel emptying, which was significantly higher than other questions (P<0.01).

Opinions on the most common symptom of STC or defecation disorders were investigated. 48% of the doctors thought that lack of defecation sensation was the most common symptom of STC, which was significantly higher than other questions (P = 0.001). 29% of the doctors thought that infrequency of defecation was the most common symptom of STC. Another 16% of the doctors thought that lumpy or hard stools was the most common symptom of STC and 6% of the doctors thought that difficult defecation was the most common symptom of STC. Of the other doctors, 1% were unclear about this definition. For the most common symptom of defecation disorders, 55% of the doctors thought that it was straining, which was significantly higher than other questions (P = 0.044).While another 40% of the doctors thought that was incomplete of bowel movement, 1% of the doctors thought that was the decreased amount of stool, another 1% of the doctors thought that was lack of defecation sensation and 3% of the doctors were unclear.

At last, we surveyed the understanding about the difference between IBS-C and FC (Table 1). 59% of the doctors thought it was abdominal discomfort or pain with constipation, pain or discomfort symptoms relieved or disappeared after defecation, which was significantly higher than other questions (P<0.001). On the other hand, we also surveyed the understanding about the difference between STC and defecation disorders. 57% of the doctors thought the difference between STC and defecation disorders was whether the patients have defecation sensation and 24% thought that it was straining, which was significantly higher than other questions (P<0.001).

### Diagnosis of constipation

**Fig 1:**
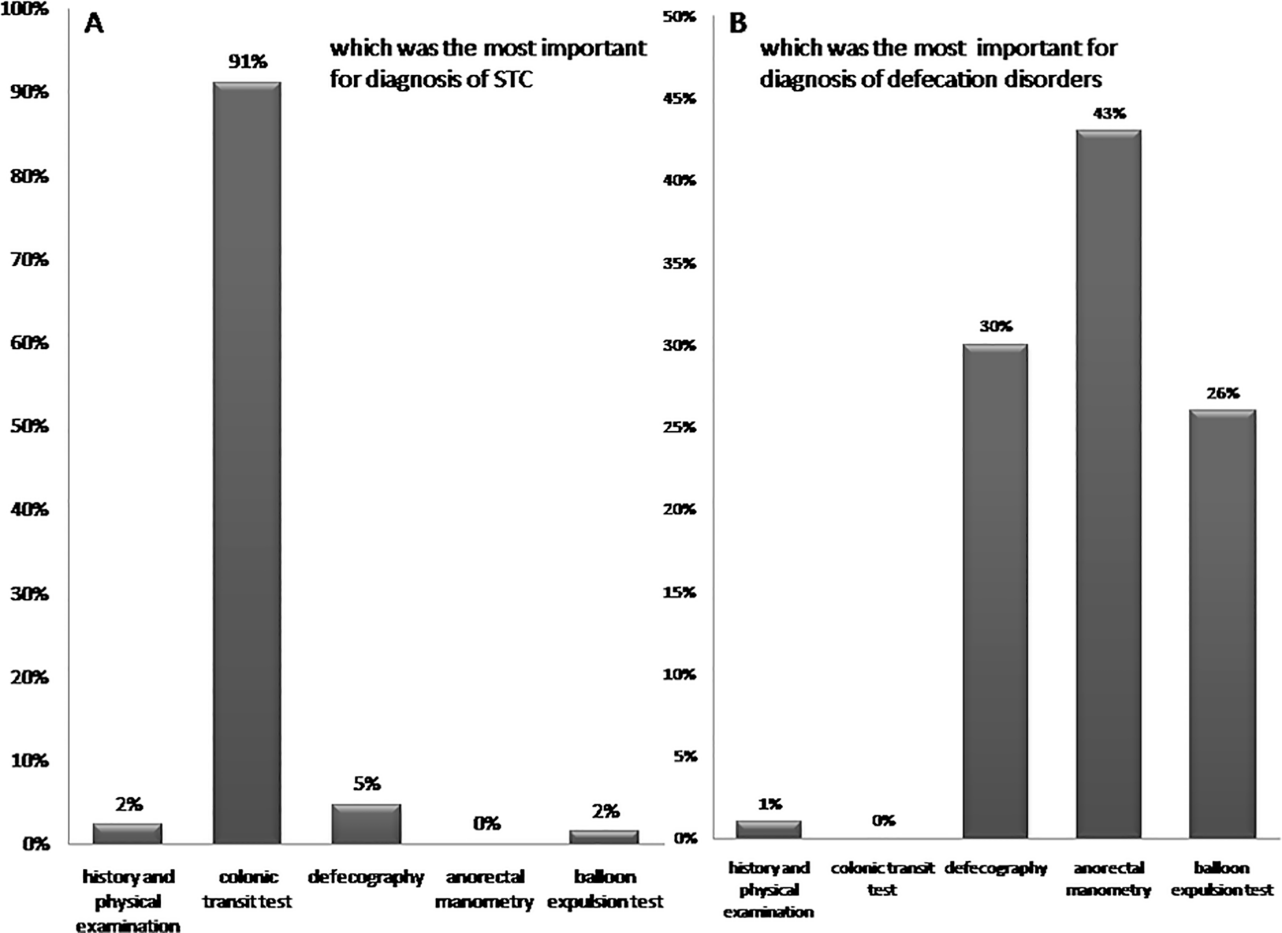
A.91% of the doctors thought that colonic transit test was most important for diagnosis STC; B. 43% of the doctors thought that anorectal manometry was the most straightforward approach to evaluate for defecation disorders

The views on which was most important for diagnosis STC were shown in Fig 1A, 91% of the doctors considered colonic transit test was most important for diagnosis STC, which was significantly higher than other questions (P<0.001). For the diagnosis of defecation disorders was shown in Fig 1B, 43% of the doctors thought that anorectal manometry (ARM) was the most important in diagnosing defecation disorders, which was significantly higher than other questions (P = 0.048).

### Management of constipation

**Fig 2:**
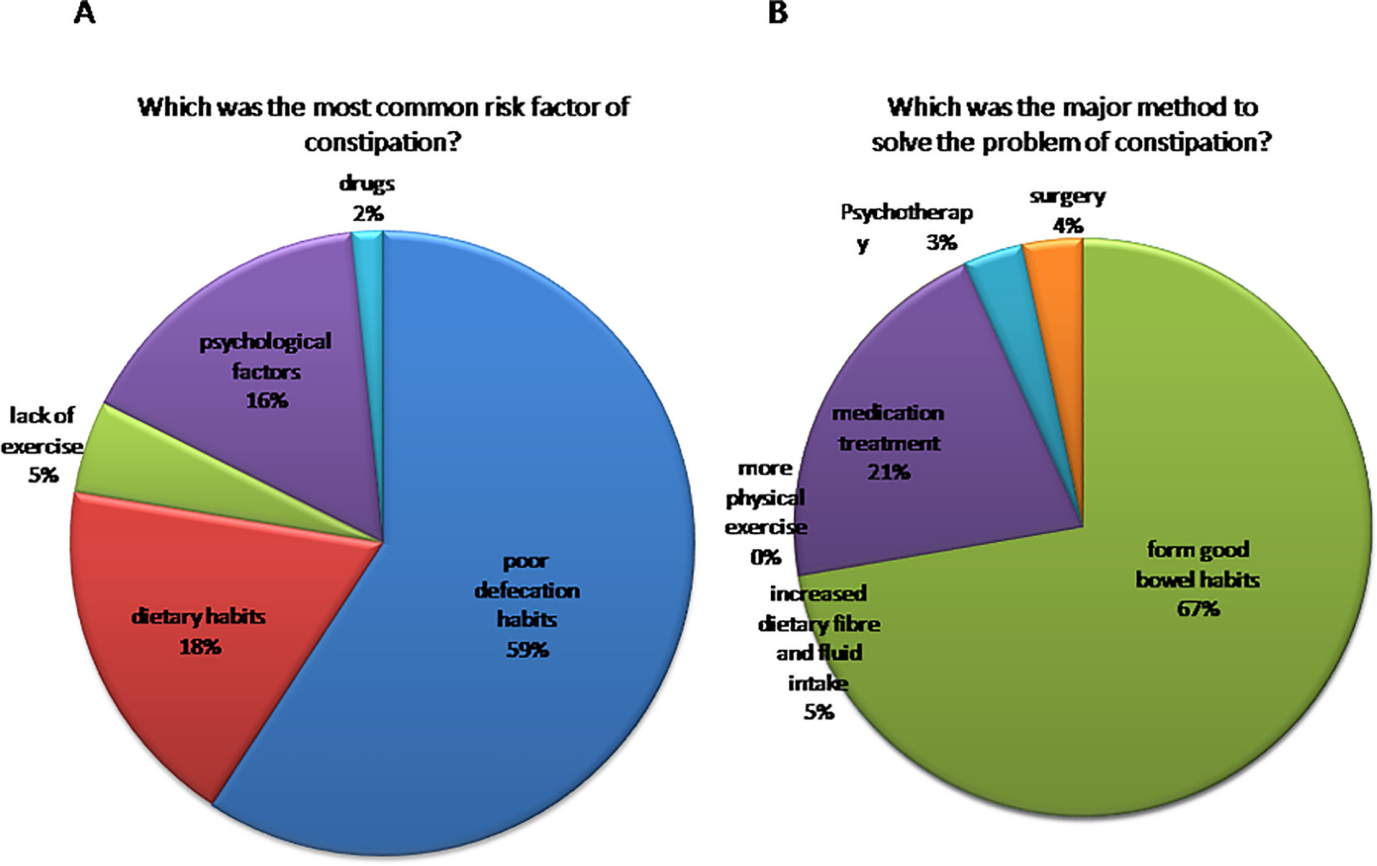
A.59% of the doctors thought that poor defecation habits were the most common risk factors of constipation; B.67% of the doctors thought that forming good bowel habits was the major method to solve the problem of constipation

First, we surveyed the most common risk factor of constipation and 59%of the doctors thought that poor defecation habits were the most common risk factors of constipation (Fig 2A),which was significantly higher than others (P<0.001). We also investigated the major method to solve the problem of constipation and 67% of the doctors thought that forming good bowel habits was the major method to solve the problem of constipation (Fig 2B), which was significantly higher than others(P<0.001).

**Fig 3:**
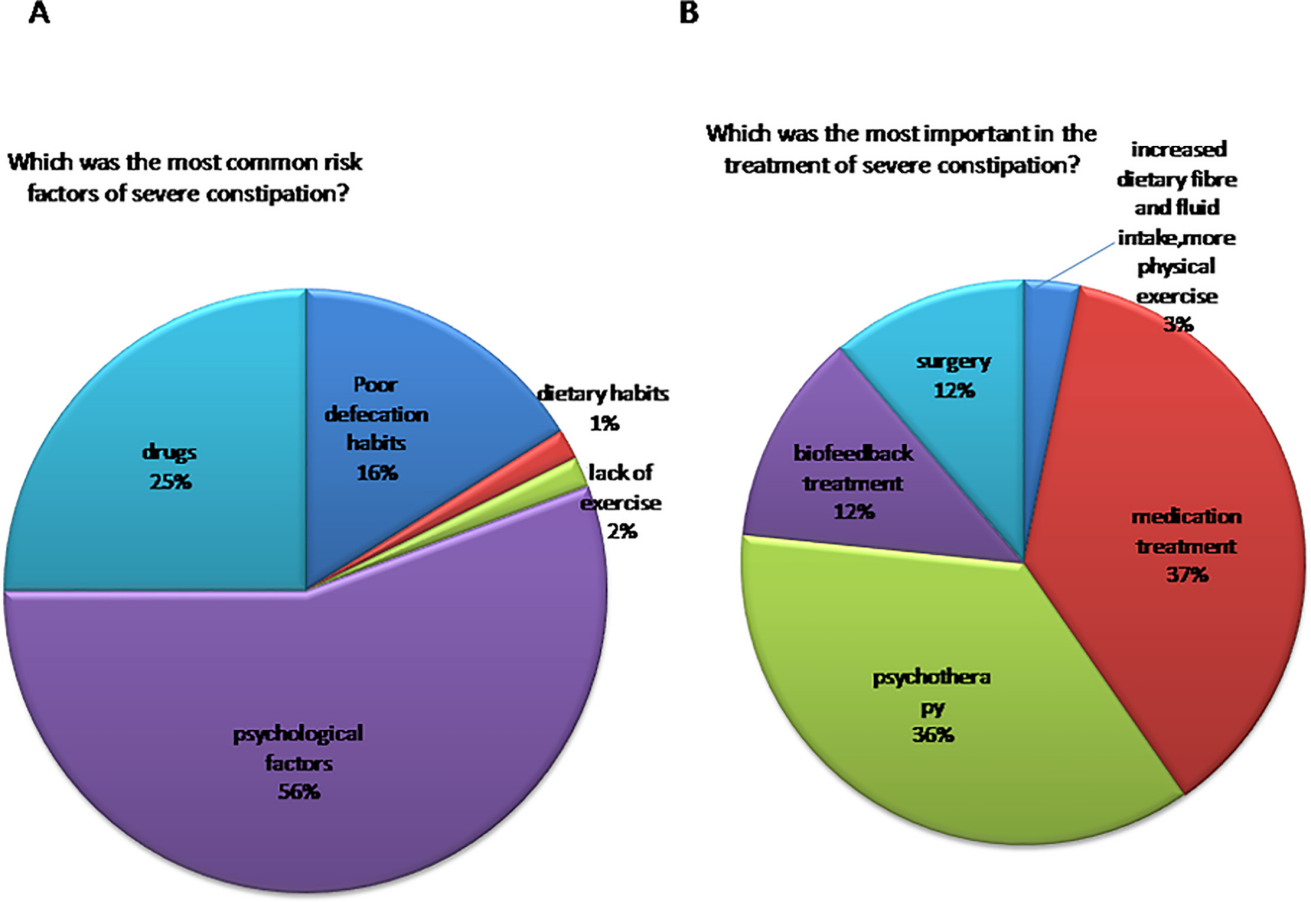
A. 56% of the doctors thought that psychological factors were the most common risk factors of severe constipation; B. There were inconsistent opinions on the treatment of severe constipation.

Second, we also studied the most common risk factors of severe constipation (Fig 3A)and 56% of the doctors thought that psychological factor was the most common risk factor of severe constipation, which was significantly higher than others (P<0.001). While, there were discordant opinions on the treatment of severe constipation (Fig 3B). Only 36% of the doctors thought that psychotherapy was most important in the treatment of severe constipation, while 37% of the doctors thought that medication treatment was most important in the treatment of severe constipation, there was no significant difference between them (P = 0.895)

At the same time, we investigated the common categories of drugs used during the process of treating constipation. 70% of the doctors recommended osmotic agents, which was significantly higher than others (P<0.001). 6% of the doctors recommended bulking agents, another 6% of the doctors recommended stimulant laxatives, 14% of the doctors recommended prokinetic agents and 4% of the doctors recommended emollients.

At last, we also surveyed the opinions about the treatment on subgroups of constipation. For the most important in the treatment of STC, 53% of the doctors suggested medication treatment(includes laxatives such as osmotic agents, bulking agents, stimulant laxatives and emollients, prokinetics, traditional Chinese medicines[31]), which was significantly higher than others (P<0.001). 16% of the doctors suggested increased dietary fibre and fluid intake, 17% of the doctors suggested more physical exercise, 2% of the doctors suggested psychotherapy and 12% of the doctors suggested biofeedback treatment. For the most important in the treatment of defecation disorders, 86% of the doctors proposed biofeedback treatment, which was significantly higher than others (P<0.001). 1% of the doctors proposed increased dietary fibre and fluid intake, 1% of the doctors proposed more physical exercise, 9% of the doctors suggested medication treatment and 3% of the doctors proposed psychotherapy.

### Differences between different working centers and the differences between different working experiences

We evaluated the differences of understanding of constipation symptoms and the diagnosis and management between medical centers and primary care centers (S1 Table). We found that most of the results were consistent. There were still some differences. 58% of the doctors who came from medical centers thought that straining was the most common symptom of defecation disorders, while only 46% of the doctors who came from primary care centers thought that straining was the most common symptom of defecation disorders, there was significant difference between the two groups (P = 0.046). 62% of the doctors who came from medical centers thought the difference between IBS-C and FC was abdominal discomfort or pain with constipation, pain or discomfort symptoms relieved or disappeared after defecation, 12% of the doctors who came from medical centers thought the difference between IBS-C and FC was abdominal discomfort or pain with constipation, pain or discomfort symptoms disappeared after defecation, while 59% of the doctors who came from primary care centers thought the difference between IBS-C and FC was abdominal discomfort or pain with constipation, pain or discomfort symptoms relieved or disappeared after defecation, and 31% of the doctors who came from primary care centers thought the difference between IBS-C and FC was abdominal discomfort or pain with constipation, pain or discomfort symptoms disappeared after defecation, there was significant difference between the two groups (P = 0.034). 36% of the doctors who came from medical centers thought that ARM was the most important in diagnosing defecation disorders, while 56% of the doctors who came from primary care centers thought that ARM was the most important in diagnosing defecation disorders, there was significant difference between the two groups (P = 0.043).

We assessed the differences of understanding of constipation symptoms and the diagnosis and management between physicians with longer than 10 years working experience and physicians with less than 10 years working experience (S1 Table). Most of the results were consistent, while there were still some differences. 55% of the physicians with longer than 10 years working experience suggested medication treatment was the most important in the treatment of STC, while only 36% of the physicians with less than 10 years working experience suggested medication treatment was the most important in the treatment of STC, there was significant difference between them (P = 0.043). 88% of the physicians with longer than 10 years working experience proposed biofeedback treatment was the most important in the treatment of defecation disorders, while only 77% of the physicians with less than 10 years working experience proposed biofeedback treatment was the most important in the treatment of defecation disorders, there was significant difference between them (P = 0.012).

## Discussion

This survey was the first study to show the understanding of constipation symptoms and the diagnosis and management of constipation by clinical physicians in China. Although most of the perceptions were consistent, there were still some different opinions. There was agreement on the most common symptoms of constipation. Majority of the attendees thought that straining was the most common symptom of constipation. However, in Western countries, studies[8, 32] defined constipation as less than three bowel movements per week and World Gastroenterology Organization[30] also defined constipation as a disorder characterized by persistent difficult or seemingly incomplete defecation, and/or infrequent bowel movements (once every 3 to 4 d or less) in the absence of alarm symptoms or secondary causes. This study may indicate that in practice in China there is a need for further study on the role of straining symptoms and there may also be a need for better establishment of consensus guidelines for constipation.

Views on the Bristol stool form as a tool to assess constipation was not consistently applied by practitioners in China. Only 34% of the doctors thought that Bristol stool form type 1 and type 2 represented constipation, which was the standard within the opinions of western countries[10]. 46% of the surveyed doctors suggested that Bristol stool form type 3 would also be considered constipation. There were some studies in other Asia-pacific regions reported that 20–50% of the patients with constipation thought Bristol type 3 stool form to be hard stool[32–35]. What’s more, Asian Neurogastroenterology and Motility Association (ANMA) also suggested Bristol types 1 to 3 stool forms as symptoms of constipation in the guidelines of chronic constipation[25]. These suggest that patients with Bristol types 3 stool forms should be carefully assessed and received proper treatment, and suggest a difference between recommendations from the East and West.

Opinions on straining were similar and most of the doctors considered increased force a requirement, with recommendation that force should be increased 26%-50%when straining. In Western countries, Kenneth W. Heaton defined straining as hold your breath and push down, in order to start passing a stool[36]. It was reported by P.R.H. Barnes[37] that electromyographic (EMG) increased by 39% when straining and R Sakakibara, et al [38] also indicated that abdominal pressure increased by 32% when straining. What’s more, Ahmed Shafik, et al[39] investigated the relationship between straining and Transverse Perineal Muscles EMG activity, with the result showing that as the increasing of straining, Superficial and Deep Transverse Perineal Muscles EMG activity increased, and Superficial and Deep Transverse Perineal Muscles EMG activity rapidly increased when abdominal pressure increased from 148cmH_2_O to185 cmH_2_O. In China, Xiao-bing Sun[40] studied the straining according to the change of anal squeeze pressure and got the following result: anal squeeze pressure of the patients who were satisfied with the biofeedback training increased by 45.6% (from 98.4mm Hg to 143.3 mm Hg). In a word, both competent views and objective test results indicate that force should be increased 26%-50% when straining and conceptions between China and Western countries were consistent.

Although 67% of the doctors defined the infrequency of defecation as less than 3 bowel movements per week when constipation, there were still 16% of the doctors defined the infrequency of defecation as times of defecation decreased compared with previously. Defecation less than 3 bowel movements per week was considered to be symptoms of constipation in Rome Ⅲcriteria[10]. While it was reported that colonic transit time ranged from 10h to 39h in Hong Kong healthy people[41]. In addition to that, patients diagnosed with constipation have bowel movements once every 2 days in India[42]. This shows that bowel movements were more frequent for people in Asia-pacific region, and defecate infrequency when constipation should be assessed according to the bowel movement characteristics of people in Asia-pacific regions instead of entirely depending on the RomeⅢ criteria.

Physicians had different opinions on duration of defecation prolonged. Only 27% of the doctors thought that it was longer than 10 minutes, while there were still a large number of doctors thought that it was longer than 20 minutes or 30 minutes. What’s more, there were many doctors thought that time of defecation became longer compared to previously bowel habits. This indicates that we need unified criteria in order to diagnose constipation precisely.

Most of the attendees thought that gastrointestinal transit test (GITT) was the most important in diagnosing STC and majority of the doctors thought that anorectal manometry, balloon expulsion test and defecography were very important in diagnosing defecation disorders. In Western countries, Prather CM[43] reported that balloon expulsion test was the most straightforward approach to evaluate for outlet obstructive constipation (OOC). And in China, Guo XF, et al.[44] reported that digital anorectal examination (DARE), gastrointestinal transit test (GITT) and ARMare effective methods of evaluating the dysmotility patterns in patients with chronic constipation. DARE and ARM could improve the diagnostic rate of OOC. Liu BH, et al[45] reported that defecography has a higher positive ratio in diagnosing OOC. It shows that both China and Western countries have consistent conceptions that examination is very important for diagnosis of different types of constipation. In our clinical practice, relevant testing should be pursued for patients with constipation and treatment should be made according to the results of the testing.

### Study limitations

The results may have been limited by the limited number physicians that participated in this study. Moreover, the comparison between different working experiences might have masked a confounding effect of inconsistent composition proportion of physicians, such as 75% of the physicians with working experience longer than 10 years, while only 25% of the physicians with working experience less than 10 years. Therefore, further research is needed in this field.

In conclusion, we were able to obtain valuable information about current views on symptoms of constipation and the diagnosis and treatment of constipation among Chinese doctors. Although most of the opinions were consistent, there were still some differences. This study indicated that in practice in China there was a need for further study on the role of constipation symptoms and there may also be a need for better establishment of consensus guidelines for constipation.

## Supporting Information

S1 Table Differences between different working centers and the differences between different working experiences. (DOCX)
