# Supplementary material for: Understanding of Constipation Symptoms and the Diagnosis and Management of Constipation in Chinese Physicians
Source: PLoS One. 2016 Mar 31;11(3):e0152801. doi: 10.1371/journal.pone.0152801 (PMC4816533; doi:10.1371/journal.pone.0152801)
Supplement: S1 Table — (DOCX) [file pone.0152801.s001.docx]

Table s1 Differences between different working centers and the differences between different working experience

|  | working experience | | | working centers | | |
| --- | --- | --- | --- | --- | --- | --- |
|  | <10years | >10years | P | medical centers | primary care centers | P |
| Which was the most common symptom of constipation? |  |  | 0.59 |  |  | 1 |
| straining | 62% | 59% |  | 61% | 61% |  |
| lumpy or hard stools | 7% | 18% |  | 17% | 15% |  |
| infrequency of defacation | 21% | 15% |  | 15% | 17% |  |
| sensation of incomplete evacuation | 3% | 4% |  | 3% | 2% |  |
| sensation of anorectal obstruction/blockage | 0% | 1% |  | 2% | 0% |  |
| Time spend at defecation prolonged | 7% | 2% |  | 3% | 4% |  |
| Which was the Bristol stool form during constipation? |  |  | 0.12 |  |  | 0.18 |
| type 1 and type 2 | 17% | 36% |  | 32% | 29% |  |
| anyone among type1 to type3 | 66% | 41% |  | 46% | 49% |  |
| anyone among type1 to type4 | 14% | 9% |  | 14% | 4% |  |
| anyone among type1 to type5 | 3% | 5% |  | 5% | 4% |  |
| anyone among type1 to type6 | 0% | 3% |  | 0% | 7% |  |
| anyone among type1 to type7 | 0% | 6% |  | 3% | 7% |  |
| How much force should be increased while straining |  |  | 0.92 |  |  | 0.31 |
| <25% | 0% | 2% |  | 0% | 4% |  |
| 25% | 10% | 13% |  | 13% | 18% |  |
| 26%-50% | 47% | 42% |  | 43% | 33% |  |
| 51-75% | 17% | 19% |  | 16% | 24% |  |
| >75% | 7% | 3% |  | 6% | 2% |  |
| unclear | 20% | 20% |  | 22% | 18% |  |
| How often was the frequency of defecation for infrequency of defecation? |  |  | 0.29 |  |  | 0.68 |
| times of defecation decreased compared with previously | 24% | 14% |  | 15% | 17% |  |
| less than 5 times per week | 0% | 2% |  | 0% | 2% |  |
| less than 3 times per week | 55% | 70% |  | 70% | 63% |  |
| less than 2 times per week | 21% | 13% |  | 15% | 17% |  |
| unclear |  |  |  |  |  |  |
| How long should be spent when duration of defecation prolonged? |  |  | 0.095 |  |  | 1 |
| >5min | 7% | 11% |  | 10% | 11% |  |
| >10min | 18% | 28% |  | 27% | 27% |  |
| >20min | 18% | 24% |  | 22% | 22% |  |
| >30min | 36% | 12% |  | 16% | 16% |  |
| time of defecation became longer compared to previously bowel habits. | 21% | 22% |  | 21% | 22% |  |
| unclear | 0% | 3% |  | 3% | 2% |  |
|  |  |  |  |  |  |  |
| Complete spontaneous bowel movements (CSBM) was defined as: |  |  | 0.55 |  |  | 0.88 |
| BM in which no laxative, enema, or suppository was used in the preceding 24 h, without a feeling of complete bowel emptying | 90% | 94% |  | 92% | 93% |  |
| BM in which no laxative, enema, or suppository was used in the preceding 24 h, which was associated with a feeling of complete bowel emptying | 3% | 3% |  | 3% | 5% |  |
| BM in which no laxative, enema, or suppository was used in the preceding 24 h | 7% | 2% |  | 5% | 2% |  |
| unclear | 0% | 0% |  | 0% | 0% |  |
| Which was the most common symptom of slow transit constipation (STC)? | |  | 0.40 |  |  | 0.52 |
| infrequency of defacation | 20% | 32% |  | 32% | 30% |  |
| lumpy or hard stools | 17% | 16% |  | 13% | 16% |  |
| lack of defecation sensation | 50% | 46% |  | 52% | 45% |  |
| difficult defecation | 13% | 4% |  | 3% | 9% |  |
| unclear | 0% | 1% |  | 0% | 0% |  |
|  |  |  |  |  |  |  |
| Which was the most common symptom of defecation disorders? | |  | 0.87 |  |  | 0.046 |
| straining | 60% | 55% |  | 58% | 46% |  |
| sensation of incomplete evacuation | 40% | 41% |  | 42% | 46% |  |
| amount of stool decreased | 0% | 2% |  | 0% | 4% |  |
| lack of defecation sensation | 0% | 2% |  | 0% | 4% |  |
| unclear | 0% | 0% |  | 0% | 0% |  |
| What was the difference between STC and defecation disorders? |  |  | 0.75 |  |  | 0.59 |
| infrequency of defacation | 3% | 7% |  | 7% | 2% |  |
| amount of stool decreased | 3% | 3% |  | 1% | 4% |  |
| lack of defecation sensation | 62% | 54% |  | 55% | 62% |  |
| difficult defecation | 28% | 24% |  | 25% | 24% |  |
| unclear | 3% | 11% |  | 10% | 7% |  |
| What was the difference between irritable bowel syndrome with constipation (IBS-C) and  functional constipation (FC)? |  |  | 0.87 |  |  | 0.034 |
| Abdominal discomfort or pain with constipation, pain or discomfort symptoms disappeared after defacation | 26% | 21% |  | 12% | 32% |  |
| Abdominal discomfort or pain with constipation, pain or discomfort symptoms relieved after defacation | 11% | 12% |  | 15% | 7% |  |
| Abdominal discomfort or pain with constipation, pain or discomfort symptoms relieved or disappeared after defacation | 59% | 57% |  | 62% | 59% |  |
| Abdominal discomfort or pain with constipation, pain or discomfort symptoms remained after defacation | 0% | 4% |  | 5% | 2% |  |
| unclear | 4% | 4% |  | 6% | 0% |  |
| Which was the most important factor for the diagnosis of STC? |  |  | 0.085 |  |  | 0.12 |
| history of present illness and physical examination | 4% | 2% |  | 0% | 7% |  |
| colonic transit test | 85% | 92% |  | 93% | 89% |  |
| defecography | 4% | 5% |  | 6% | 2% |  |
| anorectal manometry | 0% | 0% |  | 0% | 0% |  |
| balloon expulsion test | 7% | 0% |  | 1% | 2% |  |
| Which was the most important factor for the diagnosis of defecation disorders? |  |  | 0.73 |  |  | 0.043 |
| history of present illness and physical examination | 0% | 1% |  | 0% | 2% |  |
| colonic transit test | 0% | 0% |  | 0% | 0% |  |
| defecography | 28% | 31% |  | 31% | 25% |  |
| anorectal manometry | 52% | 41% |  | 36% | 57% |  |
| balloon expulsion test | 21% | 27% |  | 33% | 16% |  |
| Which was the most common risk factor of constipation? |  |  | 0.67 |  |  | 0.57 |
| poor defecation habits | 55% | 59% |  | 62% | 62% |  |
| dietary habits | 28% | 16% |  | 16% | 20% |  |
| lack of exercise | 3% | 5% |  | 7% | 2% |  |
| psychological factors | 14% | 16% |  | 15% | 13% |  |
| drugs | 0% | 2% |  | 0% | 2% |  |
| Which was the major method to solve the problem of constipation? |  |  | 0.14 |  |  | 0.37 |
| form good bowel habits | 66% | 69% |  | 72% | 60% |  |
| increased dietary fibre and fluid intake | 14% | 2% |  | 2% | 7% |  |
| more physical exercise | 0% | 0% |  | 0% | 0% |  |
| medication treatment | 17% | 21% |  | 22% | 22% |  |
| surgery | 0% | 4% |  | 3% | 4% |  |
| psychotherapy | 3% | 4% |  | 2% | 7% |  |
| Which was the most common risk factor of severe constipation |  |  | 0.71 |  |  | 0.35 |
| poor defecation habits | 14% | 18% |  | 19% | 16% |  |
| dietary habits | 0% | 2% |  | 0% | 5% |  |
| lack of exercise | 0% | 2% |  | 3% | 0% |  |
| psychological factors | 64% | 52% |  | 55% | 53% |  |
| drugs | 21% | 26% |  | 23% | 26% |  |
|  |  |  |  |  |  |  |
| Which was the most important in the treatment of severe constipation? |  |  | 0.25 |  |  | 0.97 |
| increased dietary fibre and fluid intake,more physical exercise | 0% | 4% |  | 3% | 4% |  |
| medication treatment | 28% | 38% |  | 37% | 38% |  |
| psychotherapy | 34% | 39% |  | 39% | 33% |  |
| biofeedback treatment | 21% | 10% |  | 10% | 11% |  |
| surgery | 17% | 9% |  | 10% | 13% |  |
| Which categories of drugs was the most common used during the process of treating constipation? |  |  | 0.61 |  |  | 0.33 |
| Bulking agents | 7% | 6% |  | 4% | 5% |  |
| osmotic agents | 70% | 69% |  | 74% | 66% |  |
| Stimulant | 3% | 6% |  | 1% | 7% |  |
| Prokinetic agents | 10% | 17% |  | 17% | 14% |  |
| Emollients | 10% | 3% |  | 3% | 9% |  |
|  |  |  |  |  |  |  |
| Which was the most important in the treatment of STC? |  |  | 0.043 |  |  | 0.25 |
| increased dietary fibre and fluid intake | 14% | 16% |  | 15% | 18% |  |
| more physical exercise | 25% | 14% |  | 15% | 22% |  |
| medication treatment | 36% | 55% |  | 57% | 40% |  |
| psychotherapy | 7% | 0% |  | 0% | 4% |  |
| biofeedback treatment | 18% | 14% |  | 12% | 16% |  |
| Which was the most important in the treatment of defecation disorders? |  |  | 0.012 |  |  | 0.78 |
| increased dietary fibre and fluid intake | 0% | 1% |  | 0% | 0% |  |
| more physical exercise | 0% | 1% |  | 1% | 0% |  |
| medication treatment | 23% | 4% |  | 9% | 9% |  |
| psychotherapy | 0% | 5% |  | 6% | 2% |  |
| biofeedback treatment | 77% | 88% |  | 84% | 89% |  |
